# An Unexpected Major Role for Proteasome-Catalyzed Peptide Splicing in Generation of T Cell Epitopes: Is There Relevance for Vaccine Development?

**DOI:** 10.3389/fimmu.2017.01441

**Published:** 2017-11-03

**Authors:** Anouk C. M. Platteel, Juliane Liepe, Willem van Eden, Michele Mishto, Alice J. A. M. Sijts

**Affiliations:** ^1^Faculty of Veterinary Medicine, Department of Infectious Diseases and Immunology, Utrecht University, Utrecht, Netherlands; ^2^Max-Planck-Institute for Biophysical Chemistry, Göttingen, Germany; ^3^Institut für Biochemie, Charité – Universitätsmedizin Berlin, Berlin, Germany; ^4^Berlin Institute of Health, Berlin, Germany; ^5^Centre for Inflammation Biology and Cancer Immunology (CIBCI), Peter Gorer Department of Immunobiology, King’s College London, London, United Kingdom

**Keywords:** antigen processing, CD8^+^ T cell, epitope, intracellular pathogens, proteasome, peptide splicing, vaccine

## Abstract

Efficient and safe induction of CD8^+^ T cell responses is a desired characteristic of vaccines against intracellular pathogens. To achieve this, a new generation of safe vaccines is being developed accommodating single, dominant antigens of pathogens of interest. In particular, the selection of such antigens is challenging, since due to HLA polymorphism the ligand specificities and immunodominance hierarchies of pathogen-specific CD8^+^ T cell responses differ throughout the human population. A recently discovered mechanism of proteasome-mediated CD8^+^ T cell epitope generation, i.e., by proteasome-catalyzed peptide splicing (PCPS), expands the pool of peptides and antigens, presented by MHC class I HLA molecules. On the cell surface, one-third of the presented self-peptides are generated by PCPS, which coincides with one-fourth in terms of abundance. Spliced epitopes are targeted by CD8^+^ T cell responses during infection and, like non-spliced epitopes, can be identified within antigen sequences using a novel *in silico* strategy. The existence of spliced epitopes, by enlarging the pool of peptides available for presentation by different HLA variants, opens new opportunities for immunotherapies and vaccine design.

## Introduction

**Figure 1:**
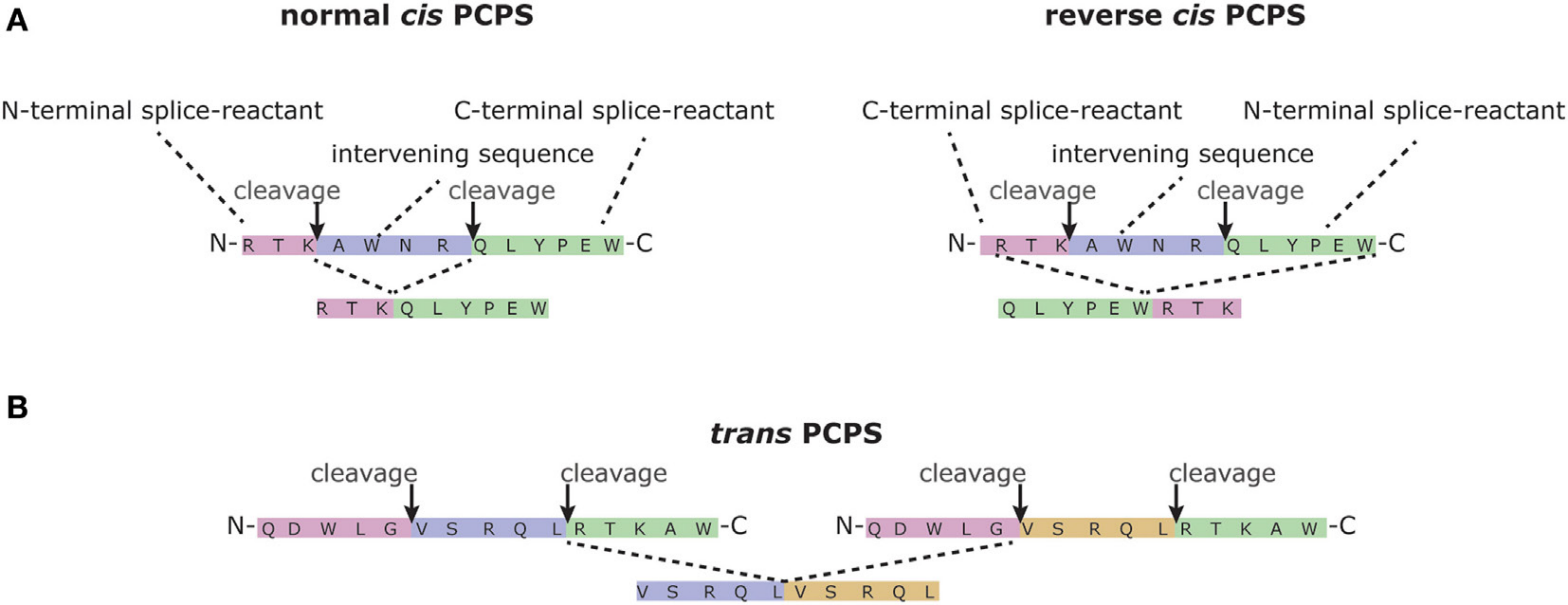
Proteasome-catalyzed peptide splicing (PCPS). PCPS may occur in different ways. **(A)** The two splice reactants can be generated by proteasome-mediated cleavage of a single peptide sequence (*cis-*PCPS), the excision of the sequence between the two splice reactants (namely the intervening sequence), followed by the ligation of the splice reactants in normal order, i.e., following the orientation from N- to C-terminus of the parental protein (normal *cis*-PCPS), or in the reverse order (reverse *cis*-PCPS). We here graphically report the formation of the spliced epitopes gp100^mel^_40-42/47-52_ (2) and gp100^mel^_47-52/4042_ (5). **(B)** PCPS can occur between two splice reactants originating from two distinct proteins (*trans*-PCPS) (6, 7). Although the latter occurs *in vitro* (6–8), its occurrence in cells is disputable (7). We here show the example of the spliced peptide gp100^mel^_35-39/35-39_, which has been identified in *in vitro* digestions of synthetic substrate by purified proteasomes (9).

Infection with most viruses and bacteria elicits a protective immune response that eradicates the pathogen and provides immunity to subsequent infections. Vaccination approaches aim to trigger similarly protective immune responses, but usually fail to activate the broad range of immune effector mechanisms reacting to natural infection. For example, most vaccines are designed and/or tested for capacity to elicit humoral responses, but intracellular pathogens, once they have entered their target cells in infected hosts, are eliminated by T cells only. Currently, the design of safe vaccines that induce protective T cell immunity, capable of monitoring new infections, remains challenging. One of the reasons is that T cells of different individuals target different pathogen-derived epitopes. Although advanced prediction programs exist for the identification of such epitopes, a larger percentage of proteins seem not to contain any epitope candidates that may trigger a protective T cell response. In this mini review, we will focus on a recently discovered mechanism underlying T cell-mediated immune recognition. Proteasomes, the proteases that generate most peptide epitopes presented to CD8^+^ T cells (1), not only degrade proteins but also paste non-contiguous sequences of a given antigen back together (2, 3). This mechanism is called proteasome-catalyzed peptide splicing (PCPS) (Figure 1). PCPS is a frequently occurring process and, importantly, expands the potential epitope pool significantly [for review, see Ref. (4)]. We here discuss the opportunities and implications of PCPS for pathogen-specific immune protection and vaccine design.

## Signaling Invaders

CD8^+^ T cells play an important role in immune protection to intracellular pathogens, including viruses and intracellular bacteria, and tumor growth. To signal infection or other intracellular aberrations, cells exploit the ubiquitin proteasome system, which safeguards the cellular proteome by degrading the majority of unfolded, immature, obsolete, and short-lived mature proteins located in the cytoplasm (1). Peptide fragments released from the proteasome may bind the transporter associated with antigen processing (TAP) for translocation into the ER. Here, they may undergo N-terminal trimming by endoplasmic reticulum aminopeptidases and be loaded into the antigen-presenting groove of MHC class I (MHC-I) molecules, if they contain an appropriate MHC-I-binding motif. Peptide loading stabilizes MHC-I molecules, which then traffic to the cell surface for display of the peptides to CD8^+^ T cells.

The proteasome, by processing most MHC-I-presented antigens, shapes the antigenic peptide repertoire available for binding to MHC-I complexes. This is illustrated by the fact that—although the antigenic peptides monitored by CD8^+^ T cells at the cell surface are influenced by the specificity of each step of the antigen presentation pathway—the two major factors selecting the MHC-I immunopeptidome are the affinity of the peptides for the cleft of the different MHC-I variants and proteasome cleavage specificity (10–13).

The proteasome can hydrolyze almost any peptide bond, but with a large range of efficiencies, resulting in huge differences in quantity between specific peptide products. One of the main cellular mechanisms to alter the peptide repertoire, produced by proteasomes, is by changing the cell’s proteasome isoform content (14). The proteasome is a multi-catalytic enzyme complex, composed of a 20S core particle, responsible for proteolysis, and different regulatory complexes, including the 19S regulatory complex, which is responsible for substrate capture and unfolding in an ATP-dependent manner [for review, see Ref. (1, 14)]. The 20S catalytic core consists of four stacked rings of seven subunits each, with catalytic activity exerted by three β subunits—i.e., β1, β2, and β5—present in the inner two rings of this particle. Under stress conditions and cytokine exposure, these subunits can be replaced by their inducible homologs LMP2/iβ1, MECL-1/iβ2, and LMP7/iβ5, leading to the formation of immunoproteasomes. Depending on cell type and levels of constitutive or induced LMP2/iβ1, MECL-1/iβ2, and LMP7/iβ5 subunit expression, cells often possess “mixed proteasomes,” which contain both standard and inducible catalytic β subunits (14). In addition, cortical thymic epithelial cells incorporate the thymus-specific β5t subunit in their proteasomes, probably to support the unique role of these cells in positive selection of CD8^+^ T cells (14–16). The exchange of the catalytic β subunits largely affects the proteolytic dynamics of the proteasome (17) and thus, depending on cytokine milieu and expressed proteasome isoforms, different peptide products will predominate among the repertoire of peptides produced. These quantitative differences in the generation of specific peptides, by the different proteasome isoforms, can mark the immunogenicity of the individual peptides, i.e., quantitative differences in epitope generation can determine whether a specific T cell response is primed, and greatly affect the immunodominance hierarchy of CD8^+^ T cells responding to infection, as demonstrated in mouse models (18–22).

## PCPS and Its Potential Relevance in CD8^+^ T Cell Response During Infection

The active site of the catalytic proteasome β subunits is formed by a threonine (T) residue at position 1 of the mature form of these enzymes. This T1 catalyses the break of the peptide bond between two residues of a substrate, thereby leading to the formation of an acyl-enzyme intermediate between the active site T1 and the N-terminal portion of the substrate. The C-terminal peptide fragment is then released. For a long period, it was assumed that the N-terminal portion of the substrate would always be released by hydrolysis. However, more than a decade ago, it was discovered that not only an H_2_O molecule but also the NH_2_-terminus of an earlier produced peptide—i.e., the C-terminal splice reactant (Figure 1)—can compete with a H_2_O molecule to attack the acyl-enzyme intermediate. This then results in a peptide bond between the N-terminal portion of the substrate—i.e., the N-terminal splice reactant—and the C-terminal splice reactant. A new, spliced peptide is thereby formed and released by the proteasome (2, 6) (Figure 1). This transpeptidation reaction is likely to be the most frequent reaction type of PCPS and was demonstrated to contribute to the generation of a handful of tumor epitopes (4). Because of the small number of spliced epitopes described in over a decade, it was believed that PCPS was a rare event of no immunological relevance. However, recent findings demonstrating that around 30% of the MHC-I immunopeptidome variety of Epstein–Barr virus (EBV)-immortalized B cell lines and primary human fibroblasts is represented by spliced peptides (23) suggested the contrary. Although it still has to be proven that this phenomenon occurs in such great extent also in other cell types, spliced peptides are likely to fulfill an immune-relevant function during the immune response against infections.

**Figure 2:**
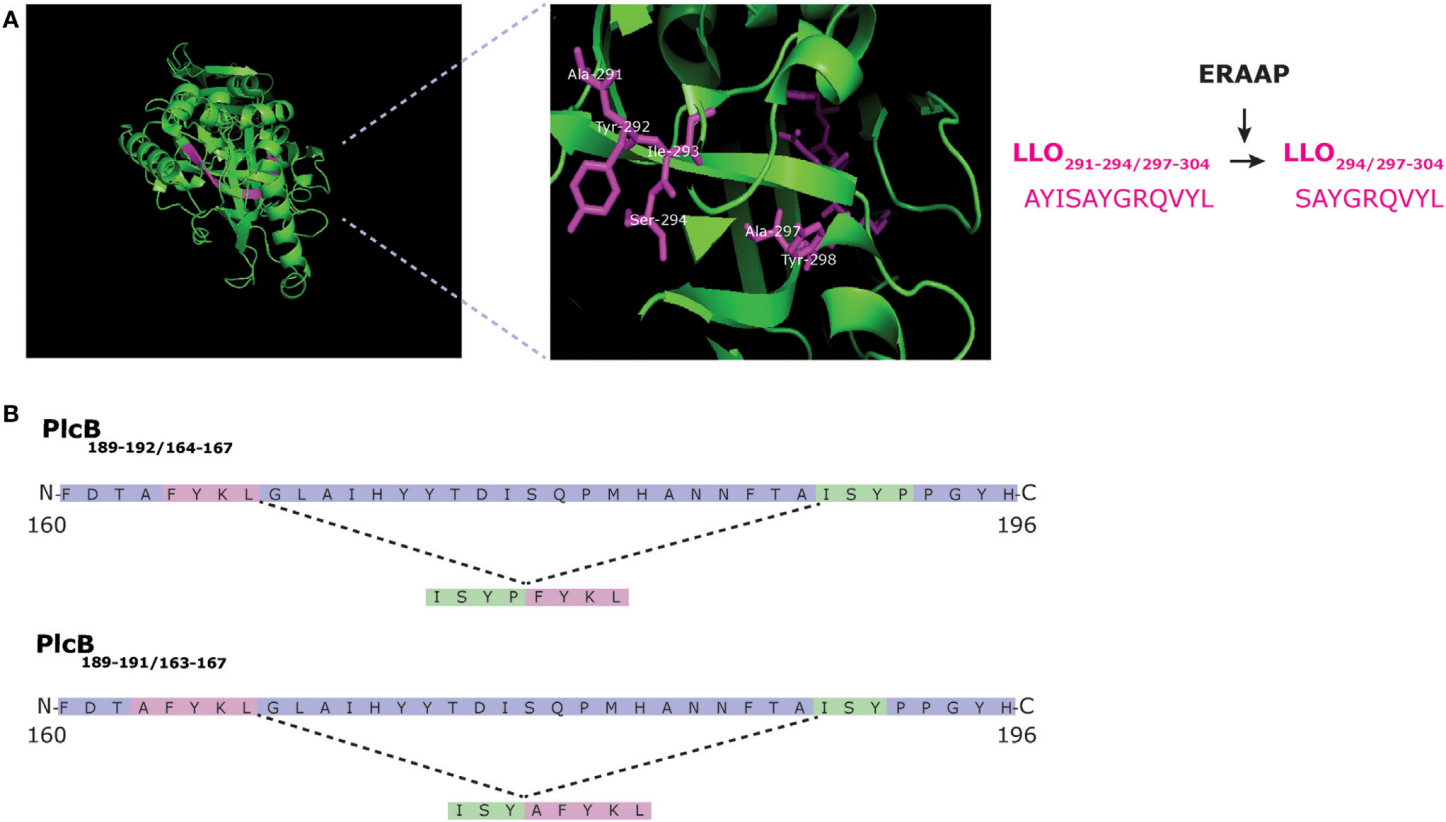
Generation of *Listeria*-derived spliced epitopes LLO_291-294/297-304_, PlcB_189-192/164-167_, and PlcB_189-191/163-167_. **(A)** Structure of LLO of *Listeria monocytogenes* with LLO_291-294/297-304_ highlighted. Proteasome-catalyzed peptide splicing (PCPS) generates the spliced epitope precursor LLO_291-294/297-304_. This peptide is N-terminally truncated by ERAP1 which results in LLO_294/297-304_ (24). **(B)** *L. monocytogenes* PlcB_189-192/164-167_ and PlcB_189-191/163-167_ are generated from the secreted bacterial virulence factor PlcB *via* reverse *cis*-PCPS (25).

First evidence supporting this hypothesis was obtained in a murine model of infection by the intracellular bacterium *Listeria monocytogenes*. We showed that CD8^+^ T cells, specific for an immunodominant epitope derived from the listeriolysin O antigen, cross-recognized an overlapping peptide produced by proteasome-mediated splicing of the LLO sequence (Figure 2A) (24). These data illustrated that CD8^+^ T cells responding to infection could recognize both non-spliced and spliced variants of the same epitope, but did not strictly prove that PCPS increased the immune-relevant epitope pool. The latter was addressed in a second study, identifying two spliced epitopes derived from the *Listeria* phosphatidylcholine-preferring phospholipase-C (Plc)B protein (25). These spliced epitopes, i.e., PlcB_189-191/163-167_ and PlcB_189-192/164-167_ (Figure 2B), triggered a specific CD8^+^ T cell response in *L. monocytogenes*-infected mice that could not be explained by any cross-reactivity toward non-spliced peptides of the same antigen or other antigens of the *L. monocytogenes*. Furthermore, mutation of the MHC-I anchor sites of these two spliced epitopes, within the PlcB antigen, abolished recognition of PlcB-transfectant cells by CD8^+^ T cells of infected mice, suggesting that the two spliced epitopes dominate the response against the PlcB antigen during *L. monocytogenes* infection (25).

These sparse data prompt the question whether spliced epitopes are just an extra pool of antigenic peptides, largely overlapping with the non-spliced peptide pool, or whether they exert a broader immune-relevant function. The fact that two spliced epitopes drive the response of the CD8^+^ T cells toward the PlcB antigen during *L. monocytogenes* infection (25) would suggest a broader function. For instance, it should be noted that various spliced epitope candidates that may be produced from *Listeria* PlcB largely exceeds that of non-spliced peptides, as calculated for both the mouse MHC-I H-2K^b^ molecule and the most frequent human MHC-I HLA-A and -B variants. In these *in silico* analyses, no non-spliced epitope candidates predicted to efficiently bind the predominant MHC-I variants were identified within the PlcB sequence, in opposite to hundreds of potential spliced epitope candidates (25). The potential immune relevance of spliced epitopes in enlarging the antigenic landscape of cells is further supported by the finding that one-third of human self-antigens is represented only as spliced peptides within the MHC-I immunopeptidomes of EBV-immortalized B cell lines and primary human fibroblasts (23). Moreover, with one-third of spliced peptides occupying one-fourth of the available MHC-I molecules, compared to two-thirds of non-spliced peptides occupying three-fourth of MHC-I molecules of the cell types investigated so far (23), the differences in quantity between spliced and non-spliced antigenic peptides may be rather small. In line with this assumption, Ebstein et al. (5) quantified the presence, at the cell surface, of gp100^mel^-derived spliced and non-spliced epitopes endogenously generated by proteasomes, using specific CD8^+^ T cell clones. Despite substantial differences between epitopes, spliced and non-spliced epitopes were presented in comparable amounts.

Thus, spliced epitopes should be equally likely to trigger CD8^+^ T cell responses as non-spliced epitopes. In addition, spliced epitopes that could be considered as variants of non-spliced epitopes, like LLO_294/297-304_, may either serve as alternative ligand for CD8^+^ T cells primed by the non-spliced epitope or, perhaps, serve as antagonists, dampening the antigen-specific CD8^+^ T cell response.

## Tools for the Prediction of Spliced Epitopes

Further studies into the immunological relevance of spliced peptides would benefit from *in silico* tools for predicting spliced epitope candidates within protein sequences of interest. However, the combinatorial possibilities of spliced peptide generation are so many that without a preliminary *in silico* strategy, any analysis would be challenging. We recently developed and tested an *in silico* approach for spliced epitope identification, using the murine model of *Listeria* infection (25). As target antigens, we selected two small, secreted virulence factors, PlcA and PlcB, and calculated all possible spliced peptides originating from these proteins. Some restrictions were introduced, based on previous studies, i.e., we selected only spliced peptides that are 8 or 9 residues long, representing the typical length of peptides presented by mouse MHC class I molecules. Furthermore, we allowed: (i) 40 residues as maximal intervening sequence length; (ii) only *cis-*PCPS, which seems to be more common than *trans-*PCPS in living cells; and (iii) that the splice reactants can be spliced in either the same or reverse order as occurring in the antigen of interest (Figure 1). The resulting reduced spliced peptide database was then analyzed using the online available SMM prediction tool for MHC-I-peptide binding affinity, to select peptides with high binding affinity for mouse H-2K^b^ molecules. Setting a cutoff for binding affinity at 16 nM, 22 spliced epitope candidates were selected and tested *ex vivo* for recognition by splenic CD8^+^ T cells of *L. monocytogenes*-infected mice. These experiments identified two spliced peptides, PlcB_189-191/163-167_ and PlcB_189-192/164-167_, as targets of the *Listeria*-specific CD8^+^ T cell response. Selecting the final spliced epitope candidates *in silico*, based on their low predicted IC_50_, showed to be the right approach since PlcB_189-191/163-167_ and PlcB_189-192/164-167_ were the two peptides, ranked as the highest affinity binders (25). Thus, we observed a correlation between predicted IC_50_ (and H-2K^b^—peptide complex stability) and priming of specific CD8^+^ T cells during infection, consistent with earlier published works on non-spliced epitopes (11–13).

Despite the positive results of the above study, the applied MHC-I-peptide-binding affinity predictions could represent a problem, because the most efficient MHC-I-peptide-binding affinity prediction algorithms have been trained on non-spliced epitope databases. PCPS and cleavage preferences significantly differ in term of substrate sequence motifs (6). Thus, it is possible that the canonical sequence motif of the non-spliced antigenic peptides bound to a given MHC-I variant differ from that of the spliced antigenic peptides. This seemed to be the case for the MHC-I immunopeptidomes of the EBV-immortalized B cell lines and primary human fibroblasts (23). Probably, a great improvement will come from studying the sequence motifs preferred by PCPS. A first attempt in that direction has been done by Berkers et al. (8). Using small peptide libraries, composed of C- and N-terminal splice reactants carrying one of two required HLA-A*02:01 anchor residues, and HLA-A*02:01 molecules coupled with a chemosensitive ligand as read out, the authors defined which residues at P2′ and P1′, and P1 and P2 position most efficiently promote PCPS. Thereby, they confirmed that rather than being a random process, PCPS is finely regulated by the substrate sequence. This specificity could be explained by the binding of the splice reactants to PCPS-binding sites of the proteasome nearby its catalytic T1 (6). However, Berkers’ approach was restricted to an epitope backbone sequence that was used as a reference and variations of that sequence were tested in terms of PCPS efficiency. It is known that not only residues in proximity but also 10 residues away may affect the cleavage-site usage by proteasomes (21, 26). Hence, the use of a fixed backbone sequence could bias the outcome of the analysis and partially restrict the results to that model sequence. Furthermore, the outcome of that pioneering work is specific for the binding motifs of HLA-A*02:01 and could thus significantly differ for other HLA-I haplotypes.

We can reckon that the development of a PCPS prediction algorithm would benefit from a sequence unbiased approach, like provided by mass-spectrometry-based identification of spliced peptides in *in vitro* digestions of synthetic polypeptides (or proteins) carried out with purified proteasomes. This approach was the basis for the definition of preferred sequence motifs of proteasome-mediated peptide hydrolysis (20, 27, 28). Such a quantitative approach, in future, may define the proteasome preferences in terms of cleavage and PCPS, and thereby facilitate further research on the immunological relevance of PCPS.

## PCPS and Cross-Reactivity

Although spliced epitope-specific T cells most likely are selected in the thymus in a similar fashion as non-spliced epitope-specific T cells, PCPS offers so far unrecognized possibilities for cross-reactivity of pathogen-specific CD8^+^ T cell responses with self-antigens. Due to the limited research into PCPS-generated pathogen-derived epitopes, such auto-reactivity has not yet been shown for spliced epitope-specific CD8^+^ T cells. However, in human T1D and mouse models of insulin-dependent diabetes mellitus, the autoreactive CD4^+^ T cell response has been shown to target *trans*-spliced self-epitopes [(29–31), and reviewed in Ref. (4)]. In addition, most spliced CD8^+^ T cell epitopes described up to date are derived from tumor-associated (self-)antigens, as discussed earlier. Thus, like for non-spliced epitopes, a spliced epitope-specific autoreactive T cell repertoire certainly exists. Given the tremendous number of possibilities for epitope splicing, there is a conceivable chance for a minor fraction of pathogen-induced spliced epitope-specific CD8^+^ T cells to display auto-reactivity.

## Relevance of PCPS for Vaccine Design

Proteasome-catalyzed peptide splicing is not a random process of ligating peptide sequences. On the contrary, preliminary evidence suggests that PCPS prefers specific peptide sequence motifs and that those motifs are not those preferred for the canonical peptide hydrolysis reaction (6, 8). This implies that the spliced and non-spliced epitope pools can differ in term of characteristics and predisposition to bind specific HLA-I haplotypes. This can explain why a large portion of self-antigens seems to be represented at the cell surface only by spliced peptides (23). The few examples of MHC-I-spliced immunopeptidomes available so far do not allow us even to speculate if those antigens represented only by spliced peptides have either a peculiar intracellular localization, specific chemical characteristics, or turnover. Moreover, we do not know whether this large representation of spliced peptide in the MHC-I self immunopeptidome occurs also in the MHC-I viral immunopeptidome. However, our preliminary evidence that the mouse CD8^+^ T cell response against a specific *L. monocytogenes* antigen (PlcB) is driven only through spliced epitopes suggests that PCPS may open novel opportunities for vaccine design.

To improve the efficacy of vaccination, especially against “difficult to treat” and rapidly mutating intracellular pathogens, vaccines need to be redesigned to elicit pathogen-specific, protective CD8^+^ T cell responses. Currently, a new generation of live, safe, recombinant vaccine vectors that encode one or multiple small antigens, derived from the pathogen of interest, is evaluated for protective capacity (32–35). Nevertheless, the extensive polymorphism of the human HLA, resulting in differing epitope-specificities and immunodominance hierarchies of responding CD8^+^ T cells in different individuals, makes it difficult to predict which antigens will provide optimal protection within a larger percentage of the population. *In silico* analysis tools are frequently used to screen antigens, but may fail to predict relevant T cell epitopes that may induce a response in context of the majority of HLA-I haplotypes, as we illustrated for *L. monocytogenes-*derived PlcB (25). Including spliced peptides as potential epitopes could aid in this, by enlarging the number of potentially immune-relevant epitopes as well as the variety of pathogen’ antigens that can be used to trigger a robust and persistent immune response. Therefore, we can speculate that further research into the immunological relevance of spliced epitopes during the immune response against infections, as well as optimization of *in silico* tools to accurately predict such epitopes within antigen sequences, can bring remarkable benefits for vaccine development.

## Author Contributions

AP drafted the manuscript, prepared the figures, and gave final approval of the version to be published. JL, MM, and AS drafted and edited the manuscript, prepared the figures, and gave final approval of the version to be published. WE edited the manuscript and gave final approval of the version to be published.

## Conflict of Interest Statement

The authors declare that the research was conducted in the absence of any commercial or financial relationships that could be construed as a potential conflict of interest.

## Abbreviations

cTECs, cortical thymic epithelial cells; EBV, Epstein–Barr virus; ERAP, endoplasmic reticulum aminopeptidase; MHC-I, MHC class I; PCPS, proteasome-catalyzed peptide splicing; PlcB, phosphatidylcholine-preferring phospholipase-C (PC-PLC); TAP, transporter associated with antigen processing.
